# Hyperechoic Content of the Fetal Colon Is Not Always Cystinuria—Case Report

**DOI:** 10.3389/fped.2021.822114

**Published:** 2022-02-23

**Authors:** Antje Knapke, Guylhène Bourdat Michel, Isabelle Marey, Pauline Le Tanno

**Affiliations:** ^1^Pediatrics, Grenoble University Hospital, Voiron, France; ^2^Pediatrics, Grenoble University Hospital, Grenoble, France

**Keywords:** antenatal, cystinuria, renal fanconi disease, congenital hyperinsulinism, *HNF4A* R76W

## Abstract

Cystinuria is a recessively inherited genetic disease causing recurrent kidney stones with risk of kidney failure. The discovery of hyperechoic colonic content on an antenatal ultrasound is considered to be a pathognomic sign of cystinuria. Herein, we present a clinical case with antenatal diagnosis of cystinuria in an ultrasound finding, which eventually revealed a multisystem disease, characterized by the association of renal Fanconi syndrome, hyperinsulinemic hypoglycemia, and hepatic dysfunction. Genetic investigations evidenced the recurrent heterozygous missense *HNF4A* (p.Arg76Trp) variant. Our case report shows that antenatal hyperechoic colonic content can hide a complex proximal renal tubulopathy, and questions the genetic counseling provided to families in the antenatal period.

## Introduction

Cystinuria is caused by dysfunction of one specific transporter in the proximal renal tubule, the di-basic amino acid (AA) transporter. It is an autosomal recessive disorder with an estimated prevalence of 1 per 7,000, characterized by mutations in the *SLC3A1* (cystinuria type a) or *SLC7A9* (cystinuria type b) genes (1). Antenatal diagnosis can be made after 20 weeks of gestation after closure of the anal sphincter, when the high concentration of cystine in the Fetal colon causes the formation of insoluble crystals. This hyperechoic colonic content is considered to be a pathognomonic sign of cystinuria (2).

Fanconi syndrome is a global dysfunction of the proximal renal tubule. Fanconi's historical description includes the renal loss of glucose, albumin, and phosphate causing rickets (3). The etiologies include a varied, congenital, or acquired version with more or less complex and severe tubular dysfunctions (4). Neonatal cases are rare. In the Quebec neonatal urinary AA screening program, only 18 cases in 2.5 million tests were identified (5). In addition, none antenatal ultrasound abnormality had ever been described in proximal tubulopathies, whereas antenatal hydramnios can be seen in distal tubular dysfunction (6).

A new complex congenital Fanconi syndrome was identified in 2010 by Flanagan and later described by Stanescu (7, 8). This syndrome is caused by a recurrent variant (p.Arg76Trp) (R76W) on the hepatocyte nuclear factor-4 alpha (*HNF4A*) gene. *HNF4A* encodes a transcription factor expressed in the intestine, proximal cells of the kidneys, islets of the pancreas, and liver. It activates a multitude of genes from Fetal life to adulthood.

Other *HNF4A* variants are known causes of neonatal hyperinsulinemic hypoglycemia (HH) and in the diabetes in the young (MODY 1) (9).

Liu et al. reviewed the phenotype of all described 15 cases harboring the recurrent p.Arg76Trp variant (10–15). All patients presented with Fanconi syndrome including nephrocalcinosis, chronic kidney failure, and short stature; all patients showed transient neonatal HH, two of them secondarily developed MODY 1, and half of them developed recurrent benign hepatic disorders. Recently, two more patients were described by Sheppard (16).

We now describe a new patient with *HNF4A* recurrent p.Arg76Trp variant with antenatal presentation mimicking cystinuria.

## Clinical Case

**Figure 1:**
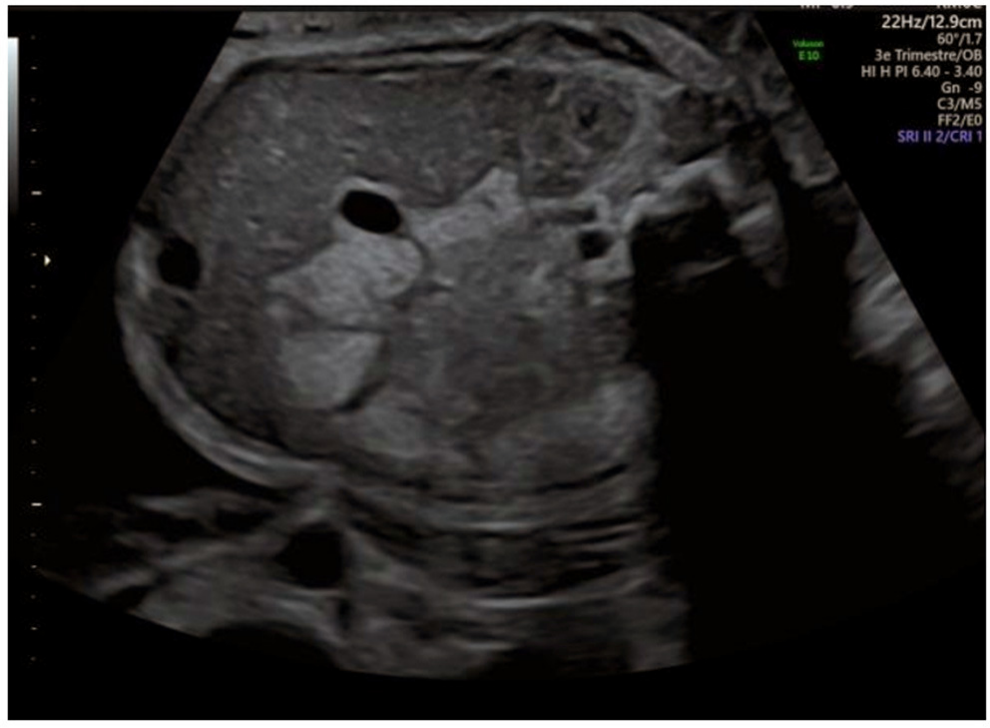
Antenatal ultrasound at 30 weeks of gestation.

A boy is expected in a non-consanguineous French family. Both parents and the 9-year-old sister are healthy. The 33-year-old mother presented gestational diabetes during her first pregnancy with no recurrence during the second. Antenatal ultrasound at 30 weeks of gestation shows isolated hyperechoic colonic content, highly suggesting cystinuria (Figure 1). Fetal growth and amniotic fluid volume are normal. The parents are informed of the cystinuria diagnosis and management, including a low risk of hypotonia-cystinuria syndrome.

The boy was born at 36 weeks of gestation with unexpected hypertrophy: 4.6 kg (>97th percentile) for 51 cm height and 35 cm head circumference (75th percentile). Adaptation is poor due to shoulder dystocia. Clinical examination at birth shows a hypotonic child, without observable anatomical birth defects. On day 3, high urinary cystine concentration is found [61 mmol/mol of creatininuria (CrU) (*N* 12–39)]. As expected, the other cationic amino acids arginine lysine and ornithine are also increased. Unexpected hyperprolinuria at 275 mmol/mol of CrU (*N* < 213) is ignored. At day 13, cystinuria reaches diagnostic values (>100 mmol/mol) with 339 mmol/mol CrU. A urine test strip shows a pH of 5 without glycosuria, proteinuria, or ketonuria. Kidney ultrasound does not show lithiasis or calcifications. The parents and the sister have normal urinary cystine levels. Hyperhydration is started to minimize urinary cystine concentrations.

Surprisingly, hypoglycemia present since birth is abnormally prolonged and requires continuous hypercaloric enteral and parenteral fluid intake up to 8.3 mg/kg/min of glucose. Laboratory results are normal for thyroid, growth hormone, and cortisol without metabolic abnormality (profile of acylcarnitine, free and total carnitine), but show inappropriate hyperinsulinemia at 28 μIU/ml (*N* < 17). The diagnosis of congenital hyperinsulinaemic hypoglycemia is established and treated by diazoxide 12 mg/kg/day.

At 3 weeks, hepatomegaly is noted, associated with high serum transaminase and alkaline phosphatase (ALP) levels: ALAT 130 IU/L (*N* < 41), ASAT 120 IU/L (*N* < 73), GGT 341 IU/L (*N* < 73), ALP 1,200 U/L (*N* < 520). Etiological search for hepatotropic viruses, autoimmunity, and alpha 1 antitrypsin deficiency is negative. Subsequently, liver enzymes decrease, while ALP increases to 2,300 IU/L.

**Figure 2:**
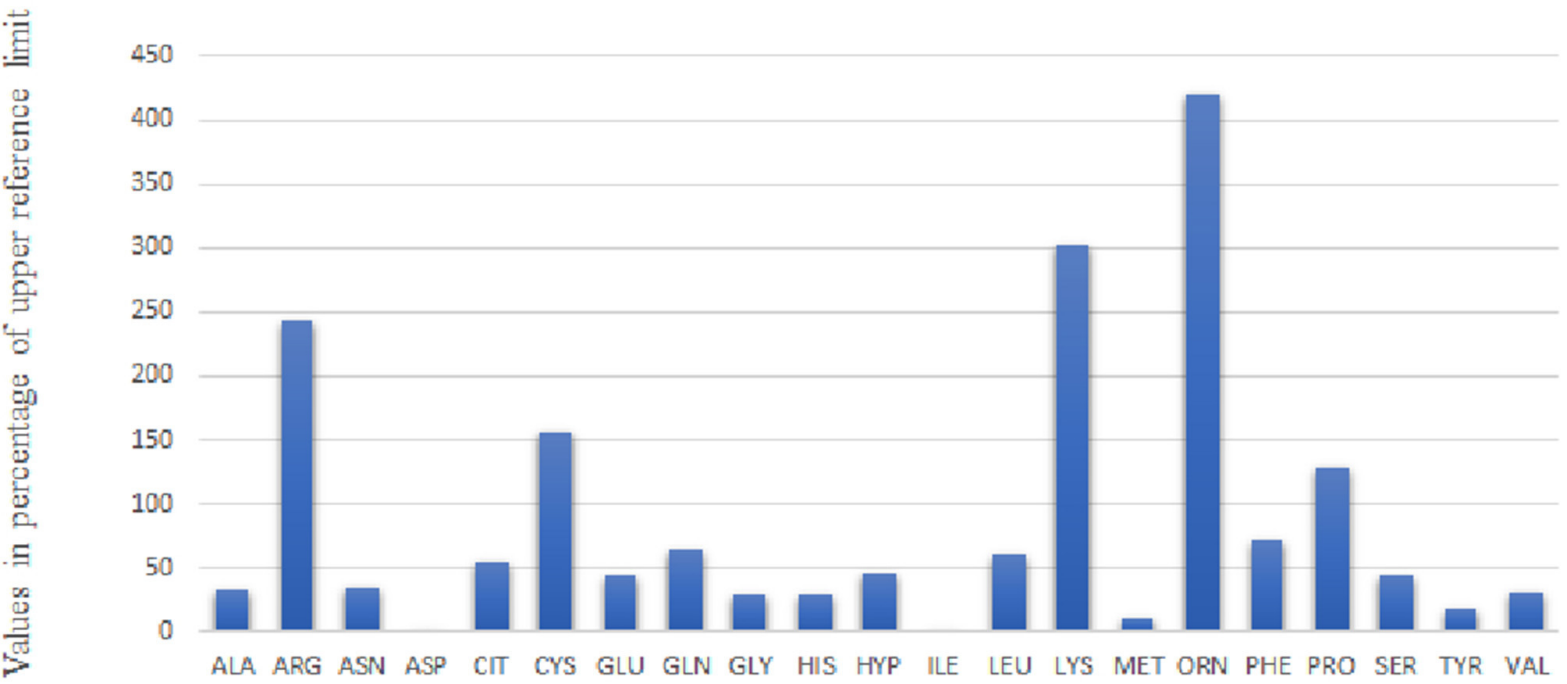
Urine Aminogram of Day 3: The values of amino acid concentration in mmol/mol CrU are given as percentage of upper reference limit adapted to age.

A thorough renal laboratory investigation shows proximal renal tubulopathy with proteinuria/CrU 825 mg/mmol (*N* < 30), beta microglobulinuria >170,000 μg/L (*N* < 3), generalized amino aciduria (Figure 2), and glycosuria 14.8 mmol/L. In addition, analyses reveal acidosis with low bicarbonatemia 17 mmol/L (*N* > 21) and hypophosphatemic rickets with typical low phosphoremia 0.8 mmol/L (*N* 0.87–1.5), low phosphate reabsorption rate at 61% (*N* > 80), high parathormone level 374 pg/mL (*N* < 50), and low vitamin 25 OHD 58 nmol/L (*N* > 75). Bone X-rays shows cupping deformation of the radial metaphysis. The child is supplemented with bicarbonate, phosphate, cholecalciferol, and calcitriol while continuing hyperhydration. At 3 months, the boy is discharged with nasogastric tube for feeding, hyperhydration, and medical treatment following a strict protocol with 2 h intakes.

Due to the unusual clinical phenotype, the diagnosis of cystinuria was questioned. The association of complex Fanconi syndrome, HH, and hepatic dysfunction triggered further etiological research. We excluded hepatorenal syndromes such as type I tyrosinemia and Fanconi Bickel syndrome. A karyotype was normal. A targeted research of the recurrent *HNF4A* (#MIM 600281) p.Arg76Trp variant, previously reported in similar presentations, was performed. Finally, at 8 months, the variant was confirmed and familial segregation showed that it had occurred *de novo*.

No other mutation was found on the 87 gene-panel of hereditary nephropathies especially no variant of *SLC3A1* (#MIM104614) and *SLC7A9* (#MIM604144) responsible for cystinuria.

At 24 months, our patient's growth follows the 50th percentile and he has normal psychomotor development. There is no sign of rickets, neither biological nor radiological. Serum and urine levels of calcium, magnesium, uric acid, and oxalate are normal. The proximal tubulopathy is characterized by a low phosphate reabsorption rate at 41%, high tubular proteinuria at 274 mg/mmol CrU, glycosuria and generalized amino aciduria with predominant cystinuric profile, and high imino acids: proline and hydroxyproline.

At 2 years of life, the kidneys are small for this age on 5th percentile without structural abnormality. Glomerular filtration rate (GFR) according to Schwartz's formula is reduced to 63 mL/min/1.73 m^2^. From the age of 17 months, the child is treated for high blood pressure with enalapril. Eating disorders persist. A gastrostomy has been in place since the age of 7 months. Glycemia is controlled by diazoxide, that could be reduced to 2.3 mg/kg/day. Liver function test is normal, but transaminases increase during each of his particularly frequent episodes of gastroenteritis.

## Discussion

We describe a new patient harboring the *HNF4A* recurrent p.Arg76Trp variant. The genetic identification enabled us to catch up the initial diagnosis of cystinuria. His phenotype was similar to those of the 17 previously described p.Arg76Trp patients (15, 16) which confirmed the high penetrance of this heterozygous point mutation. Our patient had macrosomia and neonatal onset hypoglycaemia due to hyperinsulinism. He showed hepatomegaly with transient elevated transaminases at 3 weeks with benign relapses. He developed global proximal renal tubulopathy at 51 days.

Additional clinical features are described in certain members of the 17 patients. One presented severe hearing loss (15), another hypospadias, coloboma, and mild hearing loss (16). This could either expand the phenotype of this p.Arg76Trp mutation or raise the question of genetic modifiers.

**Figure 3:**
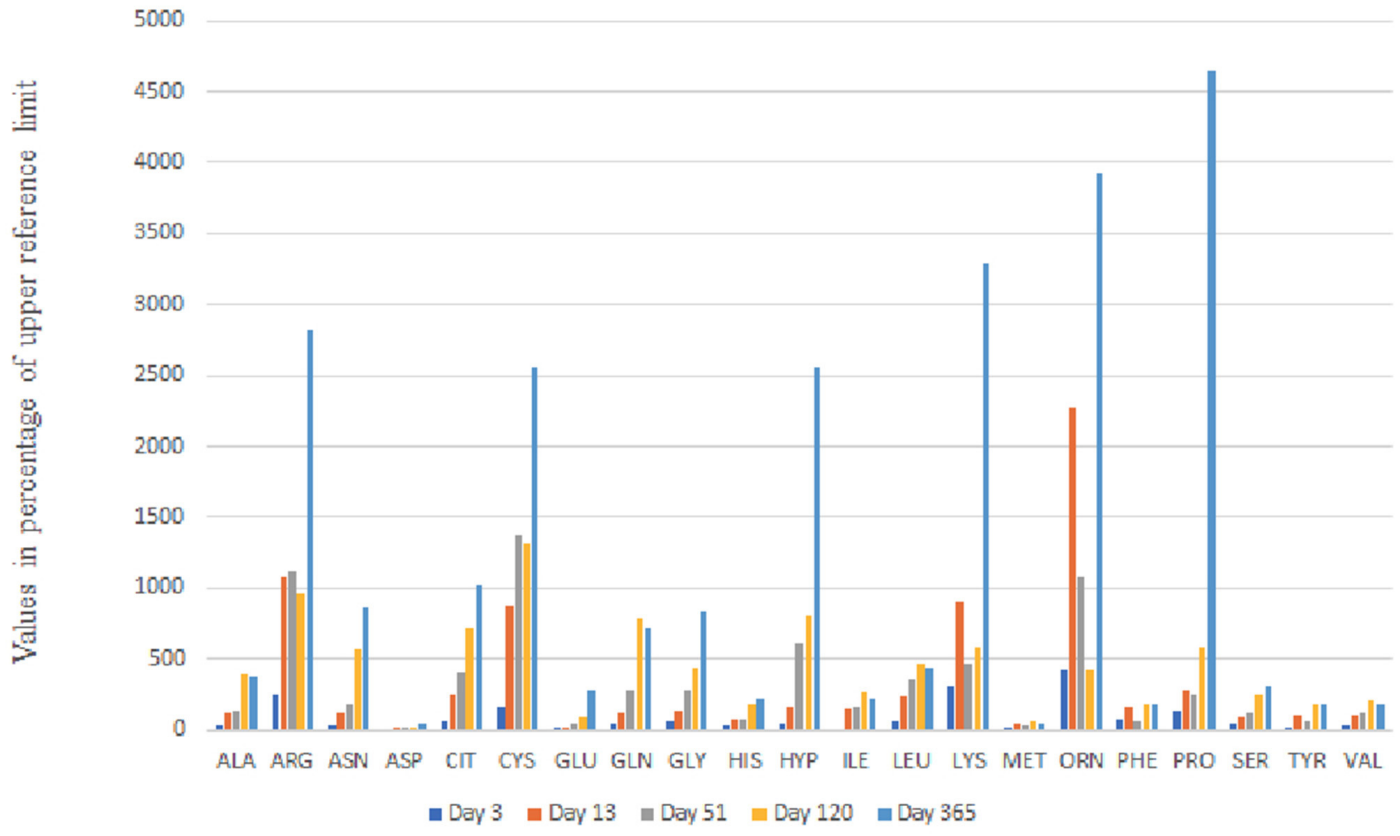
Urine Aminogram Evolution up to Day 365: The values of amino acid concentration in mmol/mol CrU are given as percentage of upper reference limit adapted to age.

The peculiarity of our patient was the neonatal onset of complete phenotype and the antenatal onset cystinuria which still dominated the urine aminogram at 1 year of age (Figure 3). We excluded classical cystinuria. There was no mutation in *SLC3A1* (#MIM104614) and *SLC7A9* (#MIM604144) genes coding for the dibasic AA transporter.

The functional impairment of this specific transporter—already suspected by antenatal hyperechoic colon content—was confirmed on the urine aminogram at day 3. It showed increased renal loss of all 4 dibasic AA including cystine (Figure 2). There was early impairment of a second transporter, the imino transporter, which explained hydroxyprolinuria and prolinuria.

However, no further dysfunction of the proximal tubule was initially seen, neither on the urine aminogram nor on the urine strip. Then, on day 51, the urine aminogram (Figure 3) revealed general amino aciduria and the laboratory investigation indicated proteinuria, hypophosphataemic rickets, and glucosuria which all pointed to a diagnosis of Fanconi syndrome.

It became clear that the impairment of the di-basic and imino AA transporter was just a forerunner sign of the complex dysfunction of the proximal tubule.

Why was our patient the only one with an antenatal cystinuria-like presentation? The onset of Fanconi syndrome in the R76W patients was generally between 4 months up to 25 years of age. Early neonatal onset of Fanconi syndrome was described in only 4 of all 17 R76W cases (10, 12, 14). These 4 cases were diagnosed in the United Kingdom where ultra sound screening is not carried out in the third trimester of pregnancy.

In order to determine whether other R76W patients had high cystinuria, we retrospectively studied the aminograms (unpublished) of five R76W cases (9, 11, 13, 14). All had late onset Fanconi syndrome. There were no common AA profiles. No cystinuric distribution was found. Only two patients had high cystine concentration (>100 mmol/mol CrU).

Renal lithiasis was not identified in any of the 17 patients. Nephrocalcinosis described in 8 of the 17 patients seems to be linked to additional renal leakage of calcium, oxalate, uric acid, and magnesium. Our patient did not present with these leakages.

The precise mechanisms of the renal dysfunction in the *HNF4A* R76W mutation remain unclear. *HNF4A* encodes a transcription factor. R76W is situated in a crucial DNA binding domain causing this unique mutation specific phenotype.

Walsh put forward the hypothesis that the *HNF4A* gene regulates the mitochondrial integrity in the kidney. This would explain the generalized tubular dysfunction as well as the abnormalities of the mitochondria on the renal biopsy of his patient (14). We postulate that moreover the R76W mutation decreases the expression of certain specific genes. Indeed, this could explain the heterogeneity of Fanconi syndrome among these patients.

Walsh's patient had a neonatal dysfunction of the specific transporter (B ° AT1) of neutral amino acids (AA) mimicking Hartnup's disease. The patient developed the complex Fanconi syndrome at the age of three (14). Stanescu described a case with decreased expression of the specific glucose transporter GLUT2. Hepatic accumulation of glycogen was found in the liver biopsy, mimicking Fanconi Bickel syndrome (8). In our case, we make the hypothesis of additional and early onset dysfunction of two transporters. A dysfunctionality of the iminotransporter and the di-basic AA transporter could explain the antenatal and persistent cystinuria/prolinuria profile.

A thorough evaluation of urinary aminograms may be a way forward to deepening our understanding of the pathophysiology of the specific R76W mutation.

For the present, our patient suffers from a diminished quality of life caused by his feeding difficulties and recurrent gastroenteritis. Long term follow-up will establish whether hyperhydration and alkalinisation can effectively prevent the development of kidney stones despite persistent high cystinuria. It will also reveal whether his chronic kidney failure will worsen, whether he'll develop short stature, whether he will be able to stop diazoxide treatment, and indeed whether he will go on to develop Mody 1 diabetes.

## Conclusion

The *HNF4A* R76W mutation is a dominantly inherited genetic disease with multi-organ involvement of high penetrance which may have an antenatal picture mimicking cystinuria. The antenatal diagnosis of cystinuria should be weighed up very carefully and include the eventuality of the development of complex tubulopathy as part of systemic disease.

## Patient Reported Outcome

The antenatal diagnosis, even though it turned out to be incorrect, allowed P. to have early medical care. For us, as parents, it was difficult to establish a relationship of trust with the doctors particularly before the discovery of his genetic mutation. The gastrostomy was a considerable help because the successive nasogastric tubes were more and more difficult for him and us to cope with. P. is very vulnerable to gastroenteritis and dehydration. The hardest things to deal with now are the continuous treatment day and night and the uncertainty about the evolution of the disease, especially kidney failure. Although ill, P. is not defined by his illness and remains a child like any other. He is a happy boy and full of life.

## Data Availability Statement

The original contributions presented in the study are included in the article/supplementary material, further inquiries can be directed to the corresponding author/s.

## Author Contributions

AK, IM, PL, and GB were involved in the conception of the paper. IM and PL were responsible for genetic research. AK and GB do nephrological follow up. AK wrote the manuscript. All authors contributed to manuscript revision, read, and approved the submitted version.

## Conflict of Interest

The authors declare that the research was conducted in the absence of any commercial or financial relationships that could be construed as a potential conflict of interest.

## Publisher's Note

All claims expressed in this article are solely those of the authors and do not necessarily represent those of their affiliated organizations, or those of the publisher, the editors and the reviewers. Any product that may be evaluated in this article, or claim that may be made by its manufacturer, is not guaranteed or endorsed by the publisher.
